# Phage “delay” towards enhancing bacterial escape from biofilms: a more comprehensive way of viewing resistance to bacteriophages

**DOI:** 10.3934/microbiol.2017.2.186

**Published:** 2017-03-31

**Authors:** Stephen T. Abedon

**Affiliations:** Department of Microbiology, the Ohio State University, 1680 University Dr., Mansfield, OH 44906, USA

**Keywords:** abortive infection systems, bacteriophage therapy, biofilm, central hollowing, native dispersion, dissemination, extracellular polymeric substances, microcolony, phage resistance, phage therapy, reduced infection vigor, seeding dispersal

## Abstract

In exploring bacterial resistance to bacteriophages, emphasis typically is placed on those mechanisms which completely prevent phage replication. Such resistance can be detected as extensive reductions in phage ability to form plaques, that is, reduced efficiency of plating. Mechanisms include restriction-modification systems, CRISPR/Cas systems, and abortive infection systems. Alternatively, phages may be reduced in their “vigor” when infecting certain bacterial hosts, that is, with phages displaying smaller burst sizes or extended latent periods rather than being outright inactivated. It is well known, as well, that most phages poorly infect bacteria that are less metabolically active. Extracellular polymers such as biofilm matrix material also may at least slow phage penetration to bacterial surfaces. Here I suggest that such “less-robust” mechanisms of resistance to bacteriophages could serve bacteria by slowing phage propagation within bacterial biofilms, that is, delaying phage impact on multiple bacteria rather than necessarily outright preventing such impact. Related bacteria, ones that are relatively near to infected bacteria, e.g., roughly 10+ µm away, consequently may be able to escape from biofilms with greater likelihood via standard dissemination-initiating mechanisms including erosion from biofilm surfaces or seeding dispersal/central hollowing. That is, given localized areas of phage infection, so long as phage spread can be reduced in rate from initial points of contact with susceptible bacteria, then bacterial survival may be enhanced due to bacteria metaphorically “running away” to more phage-free locations. Delay mechanisms—to the extent that they are less specific in terms of what phages are targeted—collectively could represent broader bacterial strategies of phage resistance versus outright phage killing, the latter especially as require specific, evolved molecular recognition of phage presence. The potential for phage delay should be taken into account when developing protocols of phage-mediated biocontrol of biofilm bacteria, e.g., as during phage therapy of chronic bacterial infections.

## 1. Introduction

As the total mass of microorganisms is thought to be similar to that of macroscopic organisms [1], microorganisms, given their smaller size, likely are the more numerous. For cellular microorganisms, in many environments, the most abundant life style is thought to be as biofilms rather than as individual planktonic cells, e.g., [2],[3]. The viruses of microorganisms [4], in turn, are thought to outnumber cellular organisms in many environments [5],[6]. Ecological interactions between what therefore are likely to be the most abundant life form, that is, viruses, and this highly prevalent state for microorganisms, i.e., as biofilms, nevertheless are appreciated at best only in broad outline [7],[8]. Here I consider the potential for otherwise “imperfect” mechanisms of bacterial resistance to bacteria-specific viruses—that is, to bacteriophages, a.k.a., phages—to aid in the survival of biofilm-associated bacteria.

Survival need not be complete to allow for subsequent organism reproduction to take place. Bodies, for example, need not remain fully intact. This concept is perhaps best appreciated in terms of herbivory in which individual plants, despite having been partially eaten, nevertheless retain some, often substantial ability to reproduce. Consistently, here I consider the potential for “imperfect” mechanisms of bacterial resistance to bacteriophages, mechanisms that are unable to outright prevent phage reproduction, nevertheless to contribute to bacterial survival. This ideally would be sufficient increases in survival to allow for at least some further bacterial reproductive success. I suggest especially that phage-biofilm interactions can represent a race between phage exploitation of biofilms, as equivalent to herbivores eating plants, and the escape especially of phage-*un*infected bacteria from biofilms—the latter as is equivalent to biofilm reproduction. Mechanisms which biofilm bacteria can employ to slow phage propagation, that is, to “*delay* the death of biofilms” [9] (emphasis added), thus could result in escape of some fraction of bacteria from attacking phages even if these mechanisms are unable to completely prevent phage replication.

**Figure 1.**
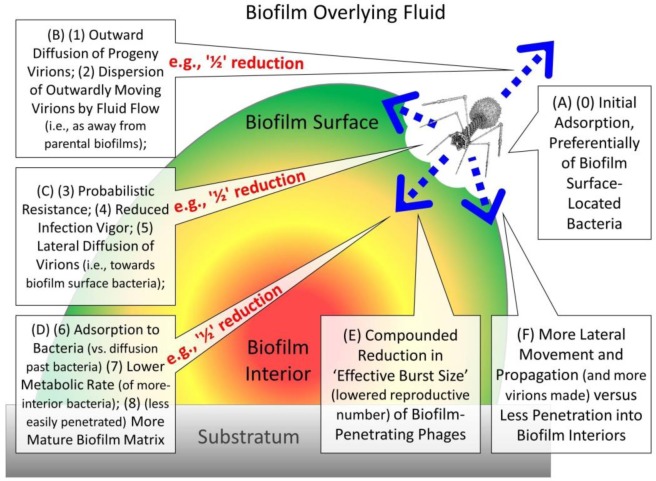
Summary of possible mechanisms of delay of virion movement, phage penetration, or phage propagation into bacterial biofilms, with a biofilm represented as a labelled, multi-colored, partial oval. Note that the shape of the biofilm as presented is not intended to provide meaning, nor the direction of phage contact or movement, except relative to the interior and surface of the biofilm. Thus, for example, phage virions could first come into contact with the top of biofilms, along the sides of “mushroom”-like projections, or along the walls of water channels which pass within a biofilm. In all cases, however, virions first make contact with a biofilm “surface” as defined in Section 3.3., i.e., the interface between extracellular matrix and overlying fluid lacking in intact extracellular matrix. Phages then, as indicated in the figure, proceed past that surface into a biofilm's “interior”, with “interior” defined as being beneath surface bacteria (also Section 3.3.). Discussion of various impediments to the penetration of phages to biofilm interiors, as indicated in Figure 1, can be found in Section 1.1. Note that the shown phage virion is not drawn to scale, i.e., it is shown as much larger than would typically be the case relative to biofilm dimensions.

Biofilm resistance to phage attack already is thought to possess a number of interesting aspects: (i) This resistance is thought to be mechanistically broader [10] than resistance by individual cells [11]–[14]. (ii) Blocks on phage propagation that are associated with bacterial death have been described as potentially altruistic [15],[16] such as towards other biofilm-associated bacteria; for the latter see also, e.g., [17],[18]. (iii) Phages may be restrained, given nutrient limitations, in their ability to fully exploit and/or lyse bacteria growing together in relatively large clumps [19]–[22]. (iv) Biofilm-attacking phage strains may *not* be present across macroscopic environments in overwhelming numbers [8],[21],[23],[24]. Building on these ideas, here I consider a number of mechanisms, as summarized in Figure 1, which individually or collectively could serve to slow phage propagation through biofilms, that is, rather than necessarily blocking phage replication altogether. The result is a framework for considering the anti-phage utility of a diversity of biofilm-bacteria structural, physiological, and dispersal strategies as well as challenges which can be associated with phage use as anti-biofilm agents.

### 1.1. Overview of mechanisms of phage delay

The various concepts indicated in Figure 1 are further introduced below, with letters and numbers corresponding to those found in the figure. Mechanisms include, with numbers in this list corresponding to subsections, (1.1.1.) reduced virion potential given diffusion alone to contact interior-located bacteria, (1.1.2.) various mechanisms which have the effect of reducing virion numbers during penetration further into biofilm interiors even given successful movement past biofilm exterior surfaces, (1.1.3.) various additional mechanisms which can have the effect of slowing phage diffusion or propagation further into the interiors of biofilms, and (1.1.4.) the idea that these mechanisms could “compound”, that is, build upon each other as anti-phage defenses. Following the provision of additional background (Sections 2.) and clarifications (Section 3.), these various issues are then discussed in sections, as indicated in the subsections found immediately below. Bacterial resistance to phages more generally is discussed in Section 4., and an introduction to especially biofilm-mediated resistance to phages is presented in Section 5. Section 6. explores further the idea of biofilm-associated resistance to phages. Section 7. considers these ideas more broadly (i.e., physiologically, evolutionary biologically, and ecologically) and in Section 8. discussion is specifically as may apply to the use of phages as anti-biofilm agents.

#### 1.1.1. Reduced virion physical association with biofilm interiors

(A) (0) Virions are assumed to preferentially initiate their acquisition of biofilms by infecting more-exterior (surface) bacteria (*sensu* Section 3.3.) versus more-interior bacteria (Section 6.1.). (B) (1) Following lysis of an initially infected bacterium, some fraction of released virions diffuse in the direction of overlying fluid (Section 6.3.2.). (2) Outwardly diffusing virions may be further drawn away from the biofilm due to overlying fluid flow (Section 6.3.2.). The net result of issues (1) and (2) is loss of some fraction of virions released from infected bacteria to away from other biofilm surface- as well as interior-located bacteria.

#### 1.1.2. Reductions in virion numbers during penetration to biofilm interiors

(C) (3) Various mechanisms of bacterial resistance to phages capable of blocking phage-infection production of new virions may function some of the time rather than necessarily 100% of the time, that is, probabilistically (Section 4.1.). (4) Various mechanisms of bacterial resistance—often in addition to probabilistic failures of infections to produce phage progeny at all—can result in reductions in phage burst sizes, and sometimes also in extended latent periods, both of which can be described as forms of reduced phage-infection vigor (Section 4.2.). (5) Among those virions released from productive phage infections which do not diffuse away from the biofilm, some fraction will diffuse laterally to infect other, more surface-located bacteria rather than towards the interior of the biofilm (Section 6.3.1.); especially surface bacteria likely represent preferred targets for phage ecological exploitation [22]. Of those virions which do not diffuse away from the biofilm, the net result of issues (3), (4), and (5) is further reduction in the number progeny virions available at a given time to penetrate more towards the interior of biofilms.

#### 1.1.3. Slowing of virion penetration to biofilm interiors

(D) (6) Virions diffusing within biofilms that consist of adsorbable bacteria will tend to encounter those bacteria, resulting in adsorption. To the extent adsorbed bacteria fail to support productive phage infections (e.g., probabilistic resistance or secondary adsorption of already phage-infected bacteria), then virions may be lost. Even given full productivity, however, adsorbed phages will spend time infecting bacteria and this is rather than diffusing past these bacteria towards more-rapid further penetration into biofilm interiors (Section 6.2.2.). (7) More-interior bacteria often will display reduced metabolic rates, including stationary phase-like physiological states. The result can be a lowered potential for phages to display robust infection vigor, i.e., as resulting in reduced burst sizes or extended latent periods (Section 5.4.), which further reduces numbers of biofilm-penetrating phages or further delays the release of virions from adsorbed bacteria, respectively. (8) There is reason to suspect that biofilm matrix as it matures and otherwise as found more towards the interior of biofilms may become less favorable to virion diffusion ([22] but also Section 4.). Of those phages penetrating more towards the interior of biofilms, issues (6), (7), and (8) should serve to further slow both virion movement and phage propagation.

#### 1.1.4. Compounding of effects affecting especially virion penetration to biofilm interiors

(E) The mechanisms discussed in the previous subsections are assumed to compound, that is, to build upon each other, with “e.g., ‘½’ reduction” as indicated in Figure 1 intended to allow visualization of such step-wise reductions. Reduction specifically is in what can be described as a phage's effective burst size [25],[26] (e.g., Section 6.2.2.), a.k.a., reproductive number [27],[28]. This is the number of virions released, per productively infected bacterium, that survive to produce more phages, here especially within the immediate vicinity (micrometers) of a parental phage infection. Thus, the likelihood for any one phage to both survive and penetrate further towards the interior of a biofilm is suggested to be reduced by ½ × ½ × ½ = 0.125, though note that the indicated “½” is an arbitrary fraction. (F) Many phage-resistance mechanisms may be *less* effective in blocking further phage acquisition of surface-located bacteria versus interfering with phage acquisition of more biofilm-interior bacteria (Section 6.3.).

## 2. Background: Biofilm-Related Biology

Introduced as background are a number of concepts concerning especially biofilm biology. These concepts are relevant to the idea that bacteria can benefit by delaying the reproduction of bacteriophages versus the presumably preferred utility of outright blocking phage reproduction. Thus, indicated by sub-section, the following concepts are discussed:

(2.1.) An organism's alleles can profit by benefiting other, particularly closely genetically related organisms, i.e., the concepts of inclusive fitness and kin selection.

(2.2.) Resistance to exploitation by other organisms need not be 100% for at least some evolutionary fitness to be retained by a resisting organism or, as driven by inclusive fitness benefits, instead by a population of both closely related and closely associated individuals.

(2.3.) Bacteria can exist as such populations, particularly as microcolonies.

(2.4.) Bacteria can “escape” from biofilms via a variety of mechanisms.

Explicit reference to these subsections, by number as indicated, is made throughout the article so that they may be readily accessed on an as-needed basis. Background concerning phage biology and phage-biofilm interactions, such as bacterial resistance to phages, is presented in sections 3, 4, and 5.

### 2.1. Inclusive fitness

With inclusive fitness, a benefit need not be realized directly by the provider of that benefit to nevertheless result in gains in Darwinian fitness [29],[30]. Both the expresser and resulting recipients of benefits, however, must carry the alleles which underlie the trait that is being selected. In this way, the costs borne by one organism may be recouped through increased reproduction by separate individuals which nonetheless carry similar genetic material. In most examples of inclusive fitness, the benefiting individuals are relatives, as with kin selection [31]. These are organisms that are more likely to carry the same alleles, contrasting randomly chosen members of the same or a different population. Further, it is particularly spatially close-by relatives which are most likely to be recipients of benefits. Indeed, physically adjacent, clonally related individuals can be in the best position to maximize inclusive fitness benefits.

From the perspective of individual organisms, we can describe the bearing of costs for the sake of helping others as *altruistic* [32]. For underlying alleles, however, self-sacrificial behaviors instead can be seen as *selfish*. That is, though reducing the fitness of allele-expressing individuals, altruistic behaviors nevertheless may enhance the “inclusive” fitness of related individuals carrying those same alleles. Alleles underlying organism self-sacrifice thus can thrive within populations so long as the alleles themselves ultimately can selfishly benefit from the sacrifice. This benefit is a consequence, literally, of close relatives in some manner helping each other. The diversity of circumstances which can select for alleles thus can be broader, or less “limited” [31], given inclusive fitness. Such inclusive fitness benefits, as noted, can be seen particularly when organisms are living within groups that are made up of genetically related individuals.

### 2.2. Incomplete resistance to exploiters

Fitness is a description of likely reproductive success, and reproductive success can decline as a function of damage to an organism's body. Consequently, mechanisms which can serve to protect an organism's body also can serve to protect an organism's fitness/reproductive ability. Protection thus can include preventing or at least mitigating “*harm*”, such as in terms of preserving health in the face of exploitation by parasites [33]. Protective mechanisms also can be viewed from an inclusive fitness perspective if we consider an organism's body as a population of closely related as well as closely physically associated individual cells. Consequently, while the display of protective mechanisms by cells making up a body can be self-sacrificial at the “lower-level” of individual cells, at a “higher-level” [34] such sacrifices can be viewed instead as altruistic to the rest of a body's cells [31].

For such actions to be self-sacrificial, then *resistance* to whatever it is that has the potential to negatively impact reproductive success cannot be absolute. Resistance, that is, cannot be both complete in its results (zero bypass) and also without negative impact of implementation (zero cost). With less than absolute resistance, then reductions in organism health and fitness can occur despite a display of resistance. That is, some cells or tissue may be lost or damaged even while other cells and tissues making up a multicellular body nevertheless survive. For example, by possessing only incompletely effective resistance mechanisms, a plant may be only partially consumed by an herbivore. The result can be retention of some reproductive ability by the partially *uneaten* plant but not necessarily as much as would be retained were the herbivore never encountered, or instead had the herbivore been both fully resisted and resisted without cost to the plant.

Retention of fitness in the face of exploitation by other organisms may be further enhanced given sacrifice by those aspects of bodies which are both replaceable and less able to directly contribute to reproduction. Contrast especially sequestered germline cells. More generally, this would be given divisions of labor between cells [35]. For example are sacrifices by neutrophilic granulocytes associated with the immune systems of our own bodies, or with other sacrificial resistance mechanisms perhaps as associated especially with modular organisms [36]. Similarly, in the case of superorganisms, such as colonies of ants or bees, somewhat sacrificial sterile worker castes will tend to provide protection to a colony rather than protection necessarily being provided by reproductive queens.

Given multiple, genetically similar cells making up a single body, or multiple individuals making up a colony of genetically related organisms, then fitness thus may be retained in the face of exploitation by other organisms. This resistance furthermore may be achieved in association with self-sacrifice by the specific resistance-effecting entities. Also relevant to the costs of displaying self-sacrifice behaviors is the concept that “there is no cost for committing suicide if the host has no chance of survival” [18], p. 4. In other words, there is little difference in terms of individual fitness between dying as a consequence of parasite action or instead dying as a consequence of resisting parasite action. Collectively, therefore, not only may organism fitness be retained in the face of partial exploitation by other organisms, but various strategies can exist such that it is especially more reproductively valuable aspects of organisms which are preserved.

### 2.3. Reproduction of bacterial microcolonies

Considered in this article are scenarios involving bacteriophages as entities which can exploit and thereby negatively impact the fitness of biofilm bacteria. To survive, biofilms must reproduce faster than they are reduced in prevalence via this exploitation, or by other antibacterial mechanisms. Biofilm “reproduction” involves the release and subsequent dispersal of constituting cells so that new biofilms may be founded [37]–[42]. Dispersal generally can be described as a sequence consisting of a departure or emigration event that is followed by a “vagrant stage” and then settling in a new location [43]. Here it is primarily departure which is of interest, which for biofilms is often described as escape, dispersion, or detachment. For biofilms to reproduce following phage attack, then at least one biofilm cell must escape to subsequently initiate a new biofilm. The more cells which escape, then presumably the greater the odds of those cells founding new biofilms. Here, though, the emphasis is not on the likelihood of individual dispersing cells initiating new biofilms but instead simply that, whatever that likelihood may be, it is collectively greater if more cells escape or, more generally, if there are more escape “events”.

If local phage propagation is stopped completely then clearly greater numbers of local, otherwise phage-susceptible cells ultimately should be able to escape. Such escape would be versus a complete local loss of all phage-sensitive cells. The latter Barraud et al. [40] would describe as “catastrophic events at one location” (p. 1), which they suggest should select for a potential to disperse from that location; see, equivalently, Andrews [36]. If phage propagation is only slowed, i.e., as via only partially effective phage-resistance mechanisms, then all local, sensitive cells ultimately could still be lost. Nevertheless, more cells may escape prior to that loss than were phage propagation not slowed. Thus, if not excessively costly to implement, then selection should favor greater dispersal potential away from localized catastrophic events through various means of delay in the impact of those events. Incomplete, “imperfect” [31], or only partial resistance by bacterial biofilms to exploiting organisms could involve sacrifice of some biofilm bacteria in combination with escape by others. This could be viewed as enhancing an inclusive fitness should sacrificed cells display resistance mechanisms along with close genetic identity to escaping cells. Incomplete resistance mechanisms presumably also can interfere with the effectiveness with which phages can exploit biofilm bacteria towards production of new phages [22]. This phage-centered perspective, however, is not emphasized here.

More precisely, what is considered is the survival and reproduction of what often are described as cellular groups or biofilm microcolonies, e.g., [2],[35],[36],[44],[45],[46]; from Costerton and Lappin-Scott [47], p. 3, “The microcolony is the basic growth unit of the biofilm and we consider bacterial growth to be sessile in nature if these microcolonies are produced…”. Furthermore, towards appreciating how microcolonies form, from Costerton et al. [48], p. 7: “Because of universal bacterial glycocalyx production in natural and pathogenic ecosystems, cells of these organisms most often divide within a hydrated exopolysaccharide matrix, so that the daughter cells are trapped in a juxtaposition that results in the formation of microcolonies of morphologically identical cells...” Microcolonies individually can represent multiple, e.g., thousands of cells [49],[50]. These cells in turn can be closely related, indeed in many cases can be presumed to be clonal [35],[48],[51],[52].

### 2.4. Mechanisms of bacterial escape

**Figure 2.**
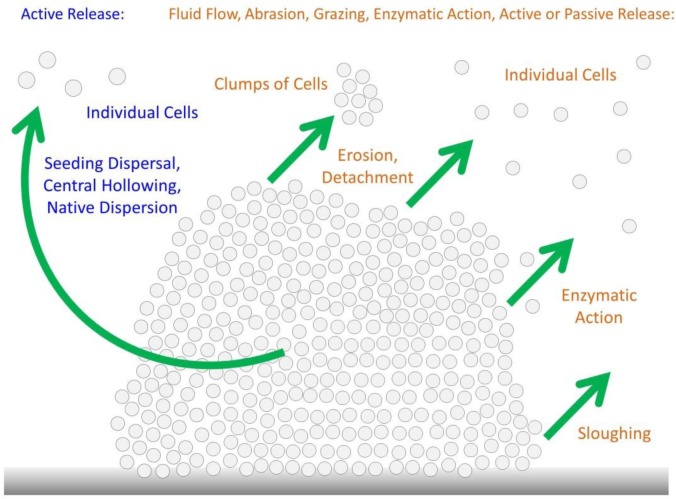
Illustration of mechanisms of bacterial escape from microcolonies. Seeding dispersal (a.k.a., central hollowing or native dispersion) is an active process, that is, following a biofilm/microcolony developmental process, as too can be erosion (a.k.a., detachment), though the latter also can and perhaps more likely occurs as a consequence of passive processes. Both processes give rise to the release of individual bacteria, but erosion also can give rise to the release of clumps of bacteria. Release can be imposed also by enzymatic action, fluid flow, abrasion, and grazing by consumer organisms (protists or animals). Sloughing is a more extensive release than simply the release of smaller cell clumps, involving instead a much more substantial portion of the biofilm. As so released, sloughing however is not shown in the figure. Though also not indicated in the figure, seeding dispersal typically will involve the escape of flagellated dispersal bacteria. Release can be more or less continuous (e.g., erosion) or instead episodic (seeding dispersal, sloughing, or as caused by grazing or abrasion).

Organisms tend to alternate between stages of dispersal and stages which are more stationary, e.g., sessile [36],[38]. Such sessility followed by dispersal followed by sessility represents, in general form, an outline of a “biofilm” life cycle [35],[37],[40],[44], or more specifically that of a biofilm microcolony. From Costerton et al. [48], p. 7: “The basic bacterial strategy is, clearly, to live within protected adherent microcolonies in nutritionally favorable environments and to dispatch mobile ‘swarmer’ cells to reconnoiter neighboring niches and to establish new adherent microcolonies in the most favorable of them.” Despite its apparent importance to biofilm reproduction, dispersal itself has been described (p. 206) as “the least-understood stage of the biofilm life cycle” [37]. This relative dearth of understanding at least in part may be because, as Kaplan further points out, also p. 206, “No single mechanism of biofilm dispersal is utilized by all bacteria.” A summary of these different mechanisms is presented in Figure 2. For further discussion, see [37]–[41],[53],[54].

#### 2.4.1. Surface erosion (detachment)

Passive release of cells from a biofilm occurs without associated changes in gene expression and otherwise cannot be blocked by knocking out specific, especially non-pleiotropically acting gene functions. Dispersal cells can be released passively from microcolonies on an ongoing basis, such as via erosion of cells from their surfaces (see Section 3.3. for definition of biofilm “surface” as employed here). It makes intuitive sense, mechanistically, for microcolonies to passively release dispersal cells especially from their exterior surfaces. This is rather than from within microcolony interiors, as via central hollowing, which is more often emphasized as a mechanism of biofilm dispersal and which also represents a non-passive mechanism of such dispersal (Section 2.4.2.). Microcolony-surface cells, that is, should have more ready access to the greater environment than more interior bacteria and directly erode into the medium surrounding the microcolony, the latter particularly without that release being intentionally initiated by the biofilm or constituting bacteria.

Surface cells, by directly interacting with the world existing outside of a biofilm, should also inherently have greater access to associated environmental resources. This includes nutrients and oxygen as found within (i) flowing fluid, (ii) viscous sublayers of fluid directly associated with surfaces, and otherwise, in the case of nutrients, (iii) as can settle or adsorb onto surfaces [44],[55],[56]. Surface cells as a consequence can display greater productivity in terms of new-cell formation than even planktonic cells [57]. Microcolony cells lost passively we thus would expect to more likely have been surface-located rather than lost from more interior portions of microcolonies. These cells also should be more metabolically active and indeed can be actively dividing. These passively released bacteria thereby can contribute to microcolony reproduction, that is, to the establishment of daughter microcolonies in new locations. Retaining this ability, even if only temporarily in the face of phage exploitation of a microcolony, therefore could serve to enhance the fitness of microcolony-generating bacteria.

#### 2.4.2. Seeding dispersal (central hollowing, native dispersion)

For microcolonies, in many cases, the formation of dispersal-stage cells is an active process, e.g., as reviewed by [37],[38]. Active refers to deliberate changes that occur within the microcolony that are associated to some degree with that subset of cells which ultimately will be released. These changes can involve the generation of flagella—for bacteria that can display motility—as well as enzymatic breakdown of biofilm matrix and/or disruption of other mechanisms of cell-to-cell adhesion [37]. Generally such active changes can be blocked experimentally by knocking out underlying gene functions, such as various enzymes or proteins that otherwise can interfere with cell-to-cell adhesion.

Active dispersal cell formation is found to occur most conspicuously within the interior of microcolonies. The result is a central hollowing of microcolonies as no-longer-aggregated interior cells are released. This release occurs over relatively short time spans into the extra-microcolony environment [37]. Active dispersal via central hollowing may be distinguished in a number of ways from passive dispersal as occurs via erosion from biofilm surfaces (Section 2.4.1.). Protection of these distinct processes we can predict may occur via subtly different mechanisms of phage resistance.

## 3. Clarifications

Six clarifications useful towards appreciating subsequent discussions are presented in this section, with a goal of heading off misinterpretation. As above, I indicate these by section number both here and in subsequent sections. In summary:

(3.1.) I am assuming that phage-resistance mechanisms don't all necessarily exist predominantly for the sake of phage resistance against specific phages.

(3.2.) While mixed-species biofilms likely are an extremely important aspect of phage-biofilm interactions, they are not explicitly considered here.

(3.3.) The concept of a biofilm “surface” is being defined explicitly in terms of phage access rather than from the perspective of biofilm or microcolony depth, shape, or orientation.

(3.4.) Consistent with the previous point, generally bacterial microcolonies are small relative to the breadth of single-species biofilms (or breadth of biofilms more generally).

(3.5.) The concept of “delay” in phage population growth is broader than just “delay” in the lysis of individual phage infections.

(3.6.) I provide an outline of the basics of phage-bacterial adsorption kinetics.

Further discussion of these ideas follows.

### 3.1. Not necessarily dedicated resistance mechanisms

**Figure 3.**
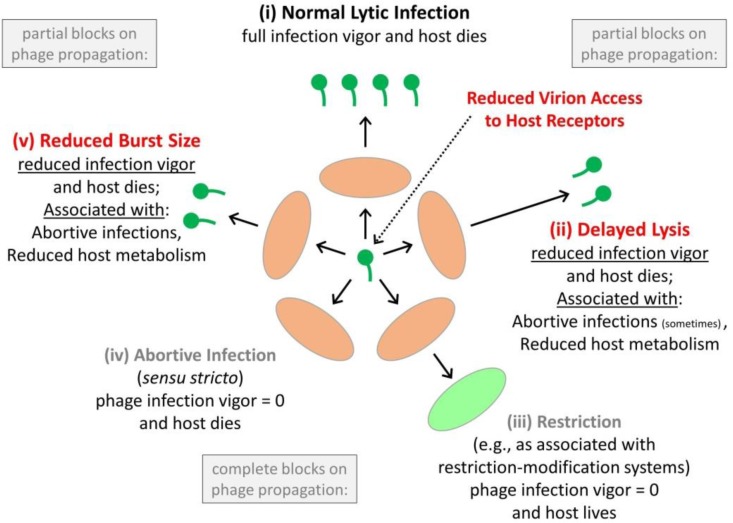
Illustration of mechanisms of bacterial resistance to phages. The inner circle of orange ovals represents phage infections of bacteria. Beyond that circle is infection outcome. This, starting at the top and going clockwise, includes (i) normal burst size and latent period, (ii) delayed lysis, (iii) no burst and bacterium survival (the latter as represented by a green oval), (iv) no burst along with lack of bacterium survival (abortive infection, *sensu stricto*), and (v) normal lysis timing but diminished burst size. Also shown (upper right) is delayed phage access to bacteria, i.e., reduced virion access to host receptors. In normal, lytic infections (top), phages display full infection vigor, meaning an outcome consisting of a typical latent period and burst size. In infections displaying reduced infection vigor, rates of phage population growth slow due to smaller burst sizes (v, left) and/or due to extended latent periods (ii, right). Alternatively, infection vigor may be reduced to zero (iii or iv, bottom). This figure is derived in part from as published in [102].

In many cases, no claims are being made, explicit or implicit, that a given biofilm property exists predominantly for the sake of protection from phage attack. As exceptions are those mechanisms which molecularly target specific phage aspects, e.g., such as strictly acting abortive infection systems (Figure 3; Section 4.2.). Instead, in many cases the suggestion is that various phenomena exist—such as biofilm structural support, chemical gradients, and bacteria dispersal mechanisms—which in addition to other roles may contribute, to certain degrees, to phage resistance. Because of their dual roles, these mechanisms may display relatively low additional costs to bacteria in comparison to more dedicated anti-phage defenses. Note that lower additional costs for phage resistance mechanisms given both multiple roles and higher densities of host bacteria has been suggested as well by Knowles et al. [58] though not necessarily in a biofilms context.

### 3.2. Explicit consideration only of single-species biofilms

Presented arguments may implicitly apply to mixed-species biofilms. What is explicitly considered, however, instead is protection from phage-mediated decimation of individual bacterial species or strains, particularly ones found clumped together into clonal cellular groups (Section 2.3.). These groups in many cases may be found in association with other, phylogenetically unrelated cellular groups, which together could make up a mixed-species biofilm. Any potential for one bacterial species to impact the phage susceptibility of another bacterial species, e.g., [59], however, is not addressed.

### 3.3. Biofilm *surfaces*

Biofilm *surfaces* are defined here exclusively in terms of phage access (italics are used in this section to indicate when usage is as defined here). That is, any portion of a biofilm which a phage particle can encounter following diffusion through a fluid—a fluid which itself does not contain intact biofilm matrix material—is considered to be a biofilm *surface*. Here this will be the case even should a *surface* line water channels within which phage virions can diffuse, but which nevertheless are considered to be located within the overall thickness of a biofilm. What is important, from the standpoint of initial virion encounters with biofilm *surfaces*, in other words, is that those encounters may initiate infections of bacteria whose overlying matrix material (i.e., glycocalyx) is in more or less direct contact with fluids that are external to that matrix material. This perspective is rather than whether on slightly larger scales the location of bacteria may or may not be described as at a biofilm's surface, but instead as “biofilm interior” [60]. The stalks of “mushroom-shaped” microcolonies thus will consist of a combination of *surface*-located and therefore more virion accessible cells as well as more *interior* cells, and here *surface* is so defined even though the stalk itself may be viewed as being found entirely within the “interior” of a biofilm.

A biofilm *surface* thereby may be viewed as a layered structure. On one side of this *surface* is matrix-free fluid within which phage virions can freely diffuse. On the other side of this *surface* is matrix/glycocalyx material. Beneath matrix material is the non-glycocalyx “surface” of biofilm-embedded bacteria. In general then, a biofilm microcolony will thus consist of some combination of such surface cells along with more interior cells, with these two locations distinguished in terms of whether phage virions can directly contact at least their overlying glycocalyx (*surface* cells) or instead phages must in some manner physically pass by or through *surface* cells in order to reach bacteria (*interior* cells). Largely due to the existence of water channels, therefore not all of the bacteria found within biofilms (“biofilm interior” cells [60]) will, here, be considered to represent *interior* cells.

### 3.4. Spatial scale of phage impact on microcolonies versus biofilms

To a large extent experimental explorations of bacteriophage-biofilm interactions have been done at spatial scales which are larger than those of individual bacterial microcolonies. Consequently, observations of localized destruction of bacterial biofilms in the course of phage propagation should *not* necessarily be equated with localized destruction of limited portions of single microcolonies. See, for example, Nale et al. [61] who observed 24-hr “plaques” in the range of 200–300 µm in diameter and which visually seem to span numerous microcolonies. Even the observations of Doolittle et al. [62]—whose reported “plaques” in fact are at least potentially on the scale of a single microcolony, i.e., ∼100 µm in diameters with numerous cells or debris remaining after 25 hr of phage propagation—could be spanning multiple microcolonies, or at least multiple cell-growth initiation points.

Rather, most of the phenomena considered here should be viewed as occurring on spatial scales of roughly 10 µm, especially, that is, spatial scales that are somewhat larger than a single bacterial cell while potentially somewhat smaller than a single microcolony. These distances are occupied by relatively few bacteria, e.g., on the order of 100 bacteria within a three-dimensional volume 10 µm in diameter. Individual microcolonies by contrast can consist of thousands of bacteria [49],[50], while single-species biofilms as commonly studied in the laboratory will tend to consist of many fold more bacteria than even that.

### 3.5. “Delay” in phage population growth

Population growth rates are controlled by a combination of “birth” rates, survival likelihood (i.e., “death” rates), and timing of reproduction. For phages, “births” correspond to virion release in the course of “bursts”, survival refers to whether released virions succeed in producing bursts of their own, and timing of reproduction here corresponds to generation times. The latter is the sum of the time until released virions (free phages) locate new bacteria to infect—which I call the phage extracellular search [63]—along with the duration of the post-adsorption phage latent period. Anything that decreases phage burst sizes, that decreases phage survival, that extends phage extracellular searches, or that extends phage latent periods will have the effect of slowing phage population growth, i.e., “delaying” a phage population's increase to certain resulting numbers or densities. It is not, however, legitimate to fully equate such delays in phage population growth with delays in phage lysis during individual infections alone, though delays in lysis can indeed contribute to delays in phage population growth.

A related issue is that phage burst sizes will tend to increase with longer phage latent periods—that is, *all else held constant*, with delayed lysis [64],[65]. Nevertheless, mechanisms exist, particularly given lower host metabolic activity, in which latent periods may be extended while at the same time burst sizes decline [66], phenomena which collectively can be described as reductions in infection vigor. Delayed adsorption, reduced survival, and reduced infection vigor independently or together thus can all result in a slowing of phage population growth. Of these mechanisms, only extended phage latent periods, however, are explicitly associated with delays in the lysis of individual phage infections, once initiated. The latter are a component of this study but nevertheless are not an emphasis.

### 3.6. Likelihood of phage adsorption

The ability of phages to impact bacteria is a function of the likelihood of phage adsorption. Generally, the more phages which are present within a given volume, then the more likely that a co-located bacterium will become phage infected. Similarly, the more phage-sensitive bacteria which are present within a volume, then the more likely that a free phage will become adsorbed. The potential for phages to impact bacteria through adsorption is, in fact, a linear function of extremely local phage densities, i.e., as over µm scales. Therefore, the more phages that are present in a bacterium's immediate vicinity then the more likely that a bacterium will become adsorbed. Equivalently, a phage released from a phage-infected bacterium that is found in the immediate vicinity of clonally related but not yet phage-infected bacteria should have a higher likelihood, per unit time, of adsorbing one of these spatially associated bacteria than will a phage that is released some greater distance away. Thus, higher local *bacterial densities* will tend to favor the adsorption of local *phages*, while higher local *phage densities* will tend to favor the adsorption of local *bacteria*.

## 4. Bacterial Resistance to Phages

Bacteria possess a number of mechanisms of phage resistance. These mechanisms are in addition to simply modifying, through mutation, those molecules that phages target. Such molecules include especially cell-surface receptors to which phage virions bind in the course of bacterial adsorption [11]–[14]. Many of these resistance mechanisms are analogous but presumably not homologous in their anti-parasite actions to those displayed by multicellular organisms, i.e., such as ourselves [67],[68]. Of recent prominence are CRISPR/Cas systems, which could be especially relevant when bacteria of specific types are present together at high densities [69], and even more recently BREX standing for Bacteriophage Exclusion [70]. Historically, however, it is restriction-modification systems as well as abortive infection systems, along with superinfection immunity [71], which have been most widely appreciated. In addition, and well known though minimally studied, is bacterial entrance into stationary phase-like states [60],[72], which at a minimum for most phages seems to interfere with the progression of phage productive infections, perhaps including phage progression into biofilms [73],[74]. This stationary-phase interference with phage infection, however, is not necessarily irreversible nor always complete [75]–[82]. In addition, contrast the ability of some phages to continue at least plaque growth on otherwise stationary phase bacterial lawns [83].

Bacteria—especially as found within biofilms—also may resist phage-mediated eradication [84] by at least partially interfering with virion diffusion via the display of extracellular polymeric substances (EPSs) [9],[60],[72],[73],[85]–[89]. See Figure 5C of [90] as possible illustration. EPS-mediated resistance may include as encoded by adsorption-interfering phage-resistance plasmids [91] or as associated with virion entrapment [92],[93]. Phage resistance within biofilms perhaps also may be effected by fully dead bacteria [73]. It is clear, however, that full inhibition of phage movement by EPS- [94], or stationary phase-associated resistance to phage infection is *not* seen with all combinations of phages, biofilms, and conditions, as various degrees of biofilm removal by phages is readily demonstrated in the laboratory [8],[21],[74],[81],[95]–[101]. This section (4.) considers especially mechanisms of bacterial resistance to phages which do not necessarily outright kill bacteria-encountering phages but nevertheless which could provide partial blocks on phage propagation (Figure 3).

### 4.1. Partial/imperfect/incomplete resistance to phages

Bacterial mechanisms of resistance to phages can be differentiated in terms of their impact on phage or bacterium viability. The interest here primarily is in such *results* of resistance mechanisms rather than how mechanistically those results are attained. From the bacterium's perspective, resistance thus can be absolute, non-existent, or somewhere in between. As our consideration primarily is in terms of resistance to productive, lytic phage infections, this means either that the phage infection is completely blocked in terms of its impact on the host bacterium (100% bacterial survival), is not blocked at all (0% survival save for resistant bacterial mutants), or instead is incompletely blocked (bacterial survival is less than 100% but more than zero). Lack of bacterial survival that results also in loss of phage activity may be described as a form of “immunity-suicide coupling” [68].

Incomplete resistance to phages is readily conceptualized as probabilistic, such that phage infections are completely blocked only some of the time. A well-known example of probabilistic resistance is seen with restriction-modification systems where occasionally phages along with their progeny are able to escape restriction [103],[104]. Indeed, any phage-killing resistance mechanism which for whatever reason does not always function fully against a given phage type may be described as probabilistic. A second scenario is resistance that results from reducing but not completely eliminating the likelihood of phage-virion adsorption to a bacterium. This can be due to lower phage affinity for receptor molecules as found on bacterial surfaces, lower display of receptors by individual bacteria, or interference with phage contact with otherwise abundant receptor molecules. In this latter case it would be phage-infection initiation which would be probabilistic. The result would be more or less the same in terms of survival of individual phage-exposed bacteria. It would be different in terms of phage survival, however, as blocks on phage infection that occur prior to phage adsorption do not necessarily result in subsequent phage inactivation.

So long as bacterial mechanisms of resistance to phages do not always result in bacterial survival, then we may describe these as only partial resistance by a bacterial population to phage attack. Bacterial display of only partial, imperfect, or incomplete resistance to attacking phages also may result in the production and release of some amount of infectious, diffusible phage progeny. Such phage production can result in greater local (e.g., Section 3.4.) bacterial losses than would be seen with full resistance.

### 4.2. Reduced infection vigor, etc.

*Phage* survival also can range from 0 to 100% following encounter with bacteria. A phage thus can survive and produce a given quantity of phage progeny 100% of the time, or neither survive nor reproduce. In addition, however, phages can survive but per productively infected bacterium reproduce to a degree that is less than what otherwise could be the case. Bacterial resistance systems, at a minimum, if successful, thus have the effect of reducing phage fitness to below 100% by reducing phage survival or, instead, given phage survival, by reducing but not necessarily eliminating phage reproductive success. These reductions in phage fitness may or may not be associated with survival of host bacteria, though survival of susceptible bacteria given phage encounter is always associated with some form of bacterial inhibition of lytic phage infection.

For our purposes, only a subset of these scenarios of anti-phage resistance is pertinent. For 0% phage survival, it is especially the case of accompanying lack of bacterial survival that has recently been of ecological interest (see abortive infections, Section 5.2.). The case of zero phage survival but 100% bacterial survival, by contrast, is expected to be beneficial to bacteria under all circumstances, at least given sufficient phage exposure [105],[106]. Though clearly important, that scenario is less of an emphasis here due to its more obvious utility to expressing bacteria. One intermediate state can be described as a reduced infection vigor [11] (see also Section 3.5.). This is seen, for example, when either the phage burst size is reduced, as often occurs in association with otherwise strictly abortive infection systems [107], or the phage latent period is extended as is occasionally seen also in association with otherwise strictly abortive infection systems, e.g., see [70]. Only the case of infections in which phage and bacterium both die, however, is described here explicitly as (strictly) abortive, i.e., as indicated in Figure 3. Thus, when abortive infection systems fail to completely block phage reproduction then phages often will display a reduced rather than completely eliminated infection vigor.

Reductions in phage burst sizes or extensions of phage latent periods, for those infections which produce phages, should result in a slowing of phage propagation through bacterial microcolonies (see Section 2.3. for introduction to bacterial microcolonies). This slowing is as compared with full-vigor displayed by that fraction of phage infections which succeed in producing phage progeny or indeed versus full vigor displayed by 100% of phage infections. This slowing also may be viewed as an example of more general scenarios of how different phenomena can impact rates of organism population growth. Declines (Section 3.5.) in population growth thus can be due to reductions in organism fecundity (which for phages is burst size), extensions in organism generation times (which for phages is controlled in part by latent period), or reductions in organism pre-reproduction survival (e.g., for phages, as due to probabilistic abortive infections). So too, rates of organism population growth should decline as a consequence of delays in the initiation of organism reproduction. For phages the latter occurs given delays in virion adsorption or due to stalled infection progression following adsorption. Reduced infection vigor along with probabilistic resistance and impediments to virion diffusion all therefore could contribute to a slowing of phage propagation, e.g., such as during phage population growth in association with a bacterial microcolony.

### 4.3. Phage “delay”

Bacteria thus possess a number of mechanisms of phage resistance including ones which can be only partially effective in preventing phage infection. I emphasize, here, that these latter mechanisms may serve to slow, that is, to delay rather than outright prevent phage population growth (Section 3.5.). Furthermore, I suggest that such delays might be observed in the course of phage propagation through a bacterial microcolony. Partial interference with phage action, in addition, may in principle be easier for bacteria to achieve towards combatting a diversity of phage types. That is, various anti-phage mechanisms can be more effective against certain phages or under certain circumstances than they are against other phage types or under different circumstances, but still not completely ineffective in these latter cases. The assumption in particular is that bacteria are not always equipped to completely block phage antibacterial activity. Nevertheless, bacteria may still possess a variety of mechanisms that in certain contexts could provide at least some degree of interference with a phage's ability to propagate through groups of, especially, related bacteria.

Significantly, there could be biases against studying, reporting on [108], or even recognizing mechanisms of bacterial resistance to phages that happen to be relatively weak in their impact—as by definition more subtle mechanisms of only phage delay may be considered. Phage delay thus can be viewed as impositions of temporary or incomplete interference with a propagating phage's access to otherwise closely associated bacteria. This delay may be imposed by a variety of mechanisms including probabilistic resistance (Section 4.1.) or incomplete resistance that results in reduced infection vigor (i.e., Section 4.2.). In addition, reduced infection vigor or indeed phage restriction (phages die, bacteria live) or abortive infection (both phages and bacteria die) may be associated with infection of bacteria that display lower metabolic rates (i.e., as considered in various sections). Furthermore, reduced rates of virion diffusion may be imposed by biofilm matrix material (ditto). Any one of these impediments when acting alone, however, may not be recognized as noteworthy. These various mechanisms, as a consequence, are not always of prominent interest in the study of bacterial resistance to phages. This proposed relative lack of scientific interest in these mechanisms may be particularly a consequence of their impact not necessarily outright blocking phage population growth such as in terms interfering with plaque formation. For example, a hypothetical mechanism which had the effect of reducing a phage's burst size by one-half (Figure 1) not only may not even be noticed but if noticed may not be deemed to be sufficiently exciting to warrant further rigorous exploration.

Notwithstanding such mechanisms not necessarily being of great interest, only partial interference especially with localized phage population growth could serve bacteria as backup mechanisms to what otherwise would be more effective means of interference with phage propagation within microcolonies. *More effective* mechanisms, that is, as may be effected by what typically are better studied as well as more virucidal mechanisms of bacterial resistance to phages [11]–[14]. Ecologically, one can envisage phages still successfully exploiting otherwise sensitive biofilm bacteria but, as a consequence of these various resistance mechanisms, doing so with less than maximal effectiveness. At the same time, bacteria may resist this exploitation, but also with less than maximal effectiveness if they are unable to outright block phage reproduction. The result would be to some degree stunted phage population growth across ecosystems, on the one hand [22], and, on the other hand, longer survival of biofilm bacteria despite local presence of phages to which they are susceptible.

### 4.4. Bacterial escape as a mechanism of phage resistance

If we constrain a bacterial population to a specific volume, or location, e.g., [18], then it is possible for bacterial mechanisms of phage resistance to have larger positive impacts on the local survival of bacteria, but smaller positive impacts on the survival of more distant bacteria. If the bacteria found within local volumes are genetically related, especially clonally related, then inclusive fitness benefits resulting from these positive effects (Section 2.1.) could select for retention of resistance mechanisms within that local population. This selection could occur, furthermore, even if the cells which are responsible for effecting the actual resistance should die in the process of resisting (Section 2.2.). If resistance mechanisms only delay phage population growth (Section 3.5.), then the utility of these mechanisms may be measured in terms of the remaining potential for bacteria to escape. Here, in general terms, this escape is associated with the potential of bacteria to disseminate away from the local volume within which phages are propagating.

Assumed for the sake of envisaging the utility of bacterial escape is that phage densities either are or will be higher within local volumes, due to localized phage population growth, than phage densities as found somewhat external to such volumes. That is, phage densities will tend to be highest within environments in the immediate vicinity, e.g., within 1 µm, of an actively lysing bacterium (Section 3.6.), but substantially lower, for example, if 100 µm of 1000 µm away. In addition, the longer or more effectively phage population growth can be delayed, then the more bacteria which may be able to escape and thereby, potentially, the greater any inclusive fitness benefits that may be associated with causing such delays. Bacterial resistance mechanisms as effected by bacterial populations thus can outright interfere with phage replication or instead can only delay phages from reaching and then killing local bacteria. The greater the number of surviving bacteria at the time or times of dissemination of individual bacteria away from a local volume, then potentially the more bacteria which may disseminate (Section 2.3.). Thus, slowing phage-mediated eradication of a spatially defined bacterial population could have utility even if local bacterial eradication is not altogether blocked (Section 2.2.). Another way of stating this is that physical movement by some fraction of bacteria away from concentrations of phages, phages that is to which those bacteria are susceptible, itself may be viewed as a form of only partial bacterial resistance to phages.

## 5. Microcolony Resistance to Phages

A problem with existing as a microcolony, versus as a separate, individual cell, is that the collective target size for phage-encounter with a cellular group should be larger than that of a single cell (see Section 2.3. for an introduction to microcolonies/cellular groups). We can speculate that a microcolony's collective target size is less than the sum of the target sizes of constituent cells as a consequence of a “shading” or “masking” of underlying cells from phage adsorption [23],[62],[84]. That is, “tight cell-cell binding may occlude phage receptors” [60] (p. 4). The collective target size of multiple cells nonetheless should still be larger than that of individual cells. A given microcolony with this larger target size should, all else held constant, be expected to encounter phages more often than individual cells will encounter phages. Phage encounter with a microcolony could result in an increased potential for phage infection of the other cells that make up the same microcolony, that is, given the proximity of these other cells to a now phage-infected bacterium [23],[73]. This increased potential for phage adsorption occurs because the highest phage densities within environments should be found in the immediate vicinity of lysing phage-infected bacteria (Sections 3.6. and 4.4.). Given these issues, we may expect that bacterial existence within microcolonies can only be stably maintained given low phage predation pressure [23], i.e., as due to inherently low phage numbers within environments [8],[21],[24] and/or as due to greater microcolony versus individual-cell resistance to phages. This section (5.) considers the latter: microcolony resistance to phages.

### 5.1. General arguments

Biofilm bacteria conceivably may be more resistant to phages than individual, planktonic cells [84],[109],[110]. Indeed, it has been suggested that bacteria may form biofilms principally as a means of evading phages [88],[111] or that increased bacterial fimbriae production might result in both phage resistance and increased bacterial propensity to form biofilms [112]. Nonetheless, data does not necessarily bear out the generalization of a hypothesis that biofilm-associated bacteria are inherently phage resistant. The basis for this doubt is that laboratory-grown biofilms often are somewhat susceptible to phages [74],[81],[97],[98]. Furthermore, there presumably are phages that exist which may be particularly effective at infecting biofilm-associated bacteria [62],[72], e.g., such as due to phage display of biofilm-matrix degrading depolymerases [21],[60],[73],[74],[81],[113]–[116]. In addition, logically it seems unlikely that phages are inherently unable to exploit to at least some degree bacteria displaying this common life style.

Rather than considering that bacteria might possess some vaguely defined potential to evade phage attack by forming into biofilms, this section (5.) considers instead precisely what those mechanisms might consist of. An underlying assumption is that these mechanisms may be more crucial to bacterial survival given existence as microcolonies rather than as individual, unassociated cells, the latter, i.e., as considered in the introduction to Section 4. [23]. Though stated within a slightly different context, Westra et al. [117] note that “organisms living together in large populations [such as clustered within microcolonies] or parasite-rich conditions [such as following phage infection of a microcolony bacterium] are more likely to evolve constitutive defenses” (bracketed asides added). With the notable exception of abortive infection-like systems, many of the mechanisms which are discussed—such as stationary phase-like physiological states, microcolony spatial heterogeneity, and EPS maturation—at least arguably are constitutively expressed. They have not, however, necessarily evolved solely for the sake of defense against phages (Section 3.1.).

### 5.2. Abortive infection systems

Even if biofilm-associated bacteria are not simply inherently phage resistant—or exhibit what can be described as a “racial” or “species” immunity [67]—they may still display mechanisms of resistance to phage infection that are, however, less absolute. Obvious examples by which biofilm bacteria but also planktonic bacteria may resist phages is through the expression of restriction-modification or CRISPR/Cas systems. Clonal clusters of bacteria, though, may display broader means of resistance to phages than individual cells. Particularly, those mechanisms in which resistance-expressing bacteria fail to survive should be unavailable to otherwise lone bacteria. What would be the point from a natural selection perspective, that is, if such a mechanism didn't actually protect expressing bacteria? With microcolonies, by contrast, it is conceivable that self-sacrificing bacteria could be helping to enhance the survival of physically adjacent clone mates (Sections 2.1.–2.3.).

The most obvious mechanism by which protective self-sacrifice would occur is through strictly acting abortive infections, that is, in which both phages and phage-infected bacteria die [10],[18],[23],[118] (Figure 3). Assuming that phage densities in surrounding environments are not very high [119], such as somewhat less than 10^7^ phages with a tropism for a given target bacterium per ml, then phage adsorption of individual bacteria making up microcolonies should not be substantial. Only minimal declines in overall microcolony fitness following phage infection, given expression of abortive infection systems, therefore may result. Thus, to the extent that microcolonies can grow faster than they are depleted in the course of experiencing periodic abortive phage infections, and especially to the point of successful escape by bacteria from those microcolonies (Section 2.4.), then a utility to possessing abortive infection systems may be realized and this is even though individual bacteria die in the process of effecting this resistance.

### 5.3. Importance of environmental phage densities

It is conceivable that microcolonies could resist phage exploitation without completely inhibiting phage replication [23],[120]. This may occur, for example, given abortive infection systems which are not fully effective in inhibiting all possibly encountered phages (Figure 3). The outcome of a lack of abortive infection system full effectiveness can include reduced phage burst sizes, described here as a form of reduced infection vigor, or delayed lysis, which also is a form of reduced infection vigor (Figure 3; Sections 3.5. and 4.2.). In addition is cell physiology-dependent means by which infection vigor can be reduced, i.e., bacteria displaying reduced metabolic activity. Also are impediments to virion movement from one location to another while in association with a single microcolony (e.g., as due to presence of EPS). A way of visualizing the utility of only partial inhibition of phage productivity is that phage propagation through microcolonies may be slowed, resulting in at least temporary phage containment. This phage delay could provide sufficient additional time in order for release of dispersal cells (Section 2.4.) to occur with some greater likelihood (Section 4.4.).

As noted (Section 5.2.), key to these mechanisms playing meaningful microcolony-protective roles is for microcolonies to be exposed to only relatively low densities of phages. Indeed, the more robust the resistance mechanism then not only the greater degree of fitness that a microcolony should be able to retain following phage exposure but also the greater the total number of phages which the microcolony may resist being exposed to. Thus, mechanisms with low failure rates in which bacteria survive given encounter with a phage should be more robustly protective then strictly acting abortive infection systems (both phage and bacterium die; Section 5.2.). Those mechanisms, in turn, should be more effective at protecting microcolonies from phages than imposition on phages, by bacteria, of reduced infection vigor or other mechanisms of phage delay, such as stationary phase-like physiologies or only partial inhibition of virion diffusion by EPS (Sections 4.2. and 4.3.).

In other words, the fewer bacteria which die following infection of a bacterium found within a clonal microcolony—zero, one, more than one, many more than one—then the more phages a group of bacteria can survive encounter with. Zero corresponds to restriction (Figure 3); one with abortive infection (*sensu stricto*; Section 4.2.); more than one occurs given some leak-through in phage reproduction despite expression, e.g., probabilistic abortive infections (Section 4.1.); many more than one with mechanisms of phage delay (Section 4.3.); and many more than one but at a faster rate should occur given no phage-resistance mechanisms at all.

### 5.4. Metabolic revival and reduced infection vigor

Phage therapy is the use of bacteriophages as antibacterial agents to treat bacterial disease (Section 8.) [121],[122]. The use of phages to experimentally treat biofilms *in vitro* is well established [8],[21],[74],[81],[95]–[101]. In the clinic, or experimentally *in vivo*, it is often less *explicitly* appreciated that biofilms have been subject to phage therapy, however. It is thought nonetheless that chronic bacterial infections often have an important biofilm component [45],[123],[124],[125]. Thus, phage treatment of chronic bacterial infections likely, to a reasonable extent, can be equivalent to phage treatment of biofilms.

A notable observation of phage treatment of chronic bacterial infections is that such treatments can take substantial amounts of time, e.g., weeks, along with a requirement for repeated phage dosing to successfully treat [126],[127],[128]. One interpretation for why this is so is that phage propagation through mature biofilms—phage reproduction in combination with virion movement—is not necessarily trivially accomplished. Indeed, it has been speculated that this treatment involves the lytic removal of a limited number of cell layers per phage dosing. That removal allows for subsequent phage access to underlying bacteria and/or metabolic revival of newly exposed bacteria, the latter potentially contributed to by nutrients released from overlying lysing bacteria [90]. This process, since it involves at a minimum phage-induced lysis of biofilm-surface bacteria, has been dubbed an active penetration of phages into biofilms [28].

Cell metabolic revival is a transition from a stationary phase-like state to a reproductive one given cell exposure to nutrients, that is, as following removal from biofilms of overlying layers of cells. Equivalent is the concept of phage-infection metabolic revival. Phages can enter states variously described as pseudolysogeny [78] or a “Hibernation' mode” [82], and to different degrees these can be followed by a phage-productive metabolic revival. Basically, phage infections can stall at some point due to low host-cell metabolic activity, but to a degree revival is possible once nutrients again become available. The result, eventually, is a phage burst. Relevant here is that such metabolic revival could convert what essentially would be non-productive phage infections, e.g., as of stationary phase bacteria, to virion-productive phage infections. This would be a transformation of what effectively are inactivated phages in terms of initial phage-infection progress to what instead are delays in the infection process, i.e., as resulting, all-told, in extended phage generation times (Sections 3.5. and 4.2.).

Such metabolic revival could be helpful to phages in terms of generating new progeny virions. Nonetheless, a delay would still be incurred. Unfortunately, it is not at all well understood what would be the duration of that delay during phage infection of biofilms. Also not well appreciated is its impact on the resulting phage burst, which we can speculate would be reduced given the starved state of the infected bacteria prior to their revival [76] (Section 3.5.). If we consider microcolonies to be multi-cell-layered structures which phages sequentially penetrate in the course of phage-productive infections, then a more or less equivalent delay may be imposed per microcolony cell layer. Thus, in short, phage adsorption to sub-surface (i.e., interior; Section 3.3.), metabolically less active or inactive cells may not necessarily result in phage death, though instead could result in some degree of reduced infection vigor.

## 6. Microcolony Spatial Resistance to Phages

This section considers how phage delay may be manifest, particularly in light of variation across microcolonies in terms of both phenotype (cell physiology and perhaps also in terms of EPS properties) and function (reproductive cells versus, potentially, protective cells). Protection, from phages, of bacterial escape from microcolonies (Section 2.4.) in terms of central hollowing (Section 2.4.2.) is contrasted with protection, from phages, of bacterial erosion from microcolony surfaces (Section 2.4.1.). An introduction to microcolonies is found in Section 2.3.

### 6.1. Microcolony heterogeneity

If microcolonies were constant across their diameters then we could predict some interesting trade-offs. For example, if all of a microcolony's constituent bacteria were in log phase, then all should be able to effectively replicate and otherwise equally contribute to microcolony structure, but so too each cell should be equivalently susceptible to phages and, for log-phase bacteria, perhaps highly susceptible. Alternatively, were all cells in stationary phase then all cells may be equivalently resistant to supporting phage infections, but so too the microcolony would not grow nor necessarily otherwise maintain itself.

**Figure 4.**
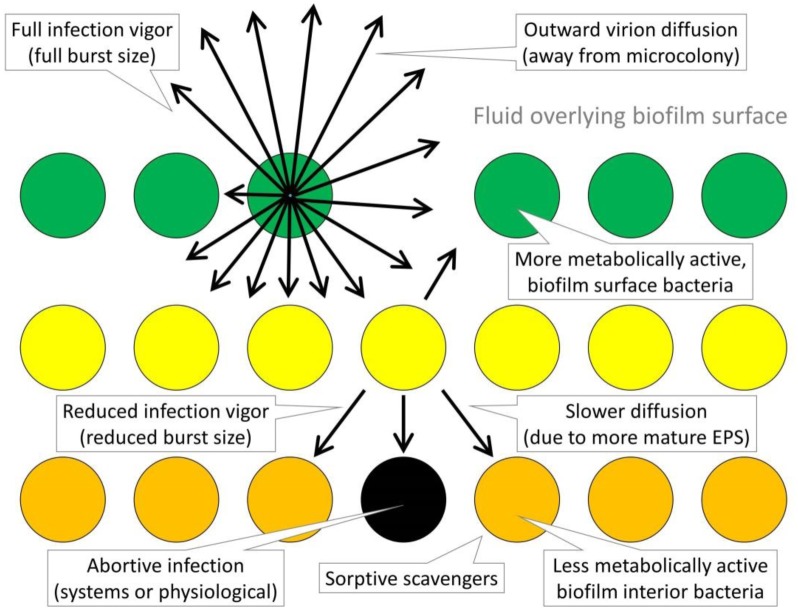
Physiological as well as physical impediments to phage propagation into microcolonies. Downward in the figure corresponds to deeper penetration into a microcolony (that is, from a microcolony's surface into a microcolony's interior, whether that surface is found on the top or side of a microcolony, or instead is found lining a water channel found within a biofilm; see Section 3.3. for clarification). Green cells (circles, top) are more metabolically active, yellow (middle) less metabolically active, and orange (bottom) even less. Arrows refer to some number of infection-released virions moving in the indicated directions. So long as bacteria are sufficiently numerous and sufficiently adsorbable then those bacteria can serve as “barriers” to deeper movement (sorptive scavenging; Section 6.2.2.), delaying phage movement in the course phage infection rather than allowing phage virions to diffuse past these bacteria. Movement may be further diminished in rate due to longer phage latent periods. Further interference with rates of phage penetration into microcolonies may include smaller burst sizes, virion diffusion other than towards the microcolony interior, and diffusion impediments imposed by EPS, the latter imposed potentially especially by more mature EPS. Reduced infection vigor can be solely a consequence of reduced bacterium metabolic rates (less infection-conducive cell physiologies) or can be due to “imperfect” expression of abortive infection-like mechanisms, or both. Strictly abortive infections (indicated with the black cell, center-bottom), even if not seen with all infections, also can have the effect of reducing progeny phage survival, thereby lowering phage effective burst sizes as well as rates of virion penetration further into microcolonies. So too can phage adsorption to already phage-infected bacteria reduce phage virion-progeny survival. The latter, though, is not explicitly indicated in the figure.

Just as trade-offs could exist in terms of the physiological states of bacteria, it has been suggested that trade-offs can be present with regard to EPS production. Higher levels of EPS production may enhance local microcolony competitive ability, that is, against neighboring microcolonies in terms of microcolony growth and survival. Such resulting greater microcolony local competitiveness, however, might come at the expense of cell dissemination ability [52]. To the extent that biofilm matrix can interfere with phage diffusion to cells making up microcolonies [9], then higher EPS production also might more effectively interfere with localized phage propagation. That ability, though, if negatively impacting the ability of bacteria to disseminate, might come at the expense of the potential for bacteria to escape from microcolonies and thereby escape from phages. Rather than considering such absolutes, however, it can be more realistic to view microcolonies as heterogeneous with regard to these various properties [129],[130], including presumably in terms of the phage susceptibility of individual cells. See Figure 4 for representation of the potential impact of such heterogeneity.

**Figure 5.**
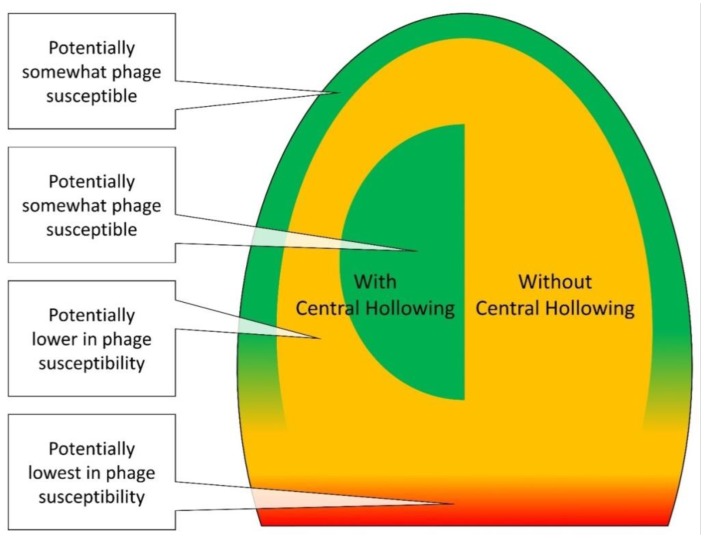
Consideration of differential phage susceptibility within a mature microcolony. To indicate all potential layers as discussed in the main text, the microcolony is depicted on its left (“With Central Hollowing”) as producing seeding-dispersal cells internally (central hollowing). Microcolonies not undergoing central hollowing (right) will not possess this additional layer. Regions consist of outer layers (green) that are other than the microcolony foundation, an interior region associated with central hollowing (also green though only as indicated on the left), a region found between these external and internal regions that potentially can serve to some degree as a phage-propagation barrier or “wall” (orange and which should be considered to continue to the center of the microcolony absent central hollowing, i.e., as indicated on the right), and also an external layer serving as the microcolony foundation (bottom, red fading into orange). As indicated previously, the overall shape of the depicted microcolony is not intended to provide information but instead solely depicts distinctions between microcolony surfaces and interiors (as clarified in Section 3.3.). Here this surface is seen as the outside of the shape especially as in contact with the outer layer of green. Microcolony interiors by contrast are seen in the figure as the inside of the shape, especially as surrounded by or instead below the outer layer of green. Surfaces found on a microcolony's top, covering its sides, or lining water channels otherwise should be considered to be equivalent—from the standpoint of this study—in terms of phage susceptibility, with microcolony interiors defined in terms of distance from any of these surfaces.

Microcolonies may be distinguished spatially particularly into areas which are more metabolically active versus less. Microcolony interiors generally are expected to be less metabolically active than exteriors [129]. We in addition can consider regions of newer growth, i.e., as associated with more metabolically active, mostly biofilm-surface-associated bacteria (see Section 3.3. for discussion of what is meant, here, by biofilm surface). Generation of active dispersal cells in the course of central hollowing should also be associated with microcolony regions possessing increased metabolic activity. Regions of attachment to underlying substratum (microcolony “foundations”) too can display physiologies that are distinct from overlying regions [39],[44],[131]. Thus, there may be as many as four more or less distinct intra-microcolony regions which could differ in their metabolic activity and thereby, potentially, in their phage susceptibility (Figure 5): (i) microcolony exteriors (i.e., surfaces; Section 3.3.), (ii) microcolony interiors (also Section 3.3.), (iii) microcolony interiors with central hollowing (Section 2.4.2.), and (iv) microcolony foundations. It is possible, therefore, that the phage potential to vigorously infect may differ depending upon a hosting bacterium's position in a microcolony. Elsewhere I have argued that the exterior of a microcolony should be more available to phage adsorption and, particularly as displaying newer growth, better able to support vigorous phage infections. Microcolony interior regions by contrast potentially possess less virion-penetrable matrix material [22], and particularly so to the extent that more mature EPS can delay phage diffusion more so than younger EPS [60].

The consequence of such heterogeneity, especially with regard to the potential for delay in phage propagation through microcolonies, is what is explored in this section 6. Important to keep in mind is that different processes can have different inherent durations, e.g., with fluid flow potentially faster than bacterial movement, bacterial movement potentially faster than phage diffusion, phage diffusion potentially faster without EPS impediments, and localized movement generally occurring over time frames that are rapid in comparison to the duration of phage infections of bacteria. Phage infections of bacteria, that is, though the means by which new virions are generated, nevertheless are not especially rapid processes, taking, e.g., multiple tens of minutes versus many tens of seconds. Phage infections can be even less rapid as well as less productive given, for example, phage infection of hosts that are experiencing lower metabolic rates [66] (Section 5.4.).

### 6.2. Central hollowing and microcolony inhomogeneity

Central hollowing, a.k.a., seeding dispersal or native dispersion (Section 2.4.2.), is a mechanism of active release of constituent bacteria from biofilms. What typically are motile bacteria (seeding-dispersal cells) form within and then escape from what literally appears as a “central hollowing” that develops in the interior of a microcolony. With central hollowing we can envisage layers in mature microcolonies as consisting, going from outside to in (Figure 5; Section 3.3.), of (1) exterior, nutrient gathering, potentially replicating bacteria (except perhaps at the foundation of microcolonies if in contact with otherwise inert substrata); (2) middle, relatively metabolically inactive, presumably microcolony strength-supplying cells in association with mature extracellular matrix (a.k.a., wall cells; see section 6.2.1.); and (3) inner, somewhat metabolically active cells that are responsible for seeding-dispersal cell formation (Section 2.4.2.). Nutrients supporting that inner-cell metabolism are potentially as supplied by autolysis of associated cells [40]. Because phage virions are more likely to first encounter cell groupings that are found on microcolony surfaces (Sections 3.3. and 6.1.), and then would need to traverse middle-layer “walls” to reach further into microcolony interiors, we have an expectation that seeding-dispersing cells actively forming in the center of microcolonies, though potentially metabolically able to support vigorous phage infections and phage population growth, nevertheless may be inherently less available to do so as a consequence of lowered potential for phage access. Of interest, in terms of phage delay, therefore is both whether and how long these “walls” may be able to protect such inner dispersal-cell “nurseries” from phage access.

#### 6.2.1. Central hollowing supporting walls

Central hollowing (Section 2.4.2.) tends to occur only as microcolonies reach sizes of 40 to 80 µm in diameter [39],[132]. Such a size presumably is wide enough for the microcolony as a whole to retain mechanical strength despite central hollowing. These microcolonies thereby may retain a still-aggregated, supporting, multicellular exterior structure (wall) that is thick enough to resist failure prior to the point of its breaching to release matured dispersal cells from microcolony interiors. Indeed, these microcolony walls not only can consist of visually distinct structures (“shell-like”) but also can possess a consistent thickness [132]. Mechanisms must exist which serve to prevent the disruption of EPS and/or cell-to-cell adhesion within microcolony walls, i.e., so as to maintain resistance by these walls to shear stresses. One mechanism may simply be time, as the portion of microcolonies surrounding the developing dispersal cells can thin over the course of central hollowing as internal volumes increase, that is until failure of the surrounding wall structure allows active dispersal cells to escape [39].

**Figure 6.**
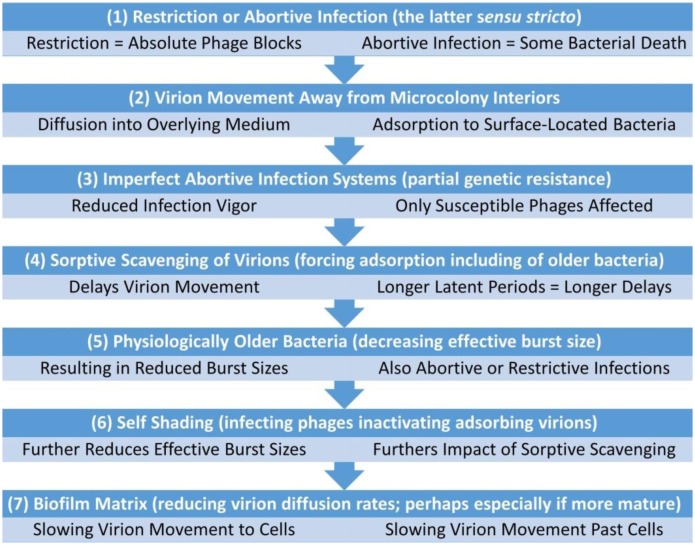
Summary of phenomena potentially interfering with phage penetration or propagation towards microcolony interiors. These phenomena are discussed as follows: (1) Mechanisms with specific molecular targets are often not present or at least not fully active against specific phage types, but may block phage propagation altogether if present. (2) Viruses that are released from infections even if displaying full vigor nevertheless likely are not available *solely* for penetration into microcolony interiors. (3) If present, resistance mechanisms may display less than full activity against phages, acting probabilistically and potentially resulting in infection vigor that is reduced though not to zero. (4) To the extent that infection of bacteria can impose delays on virion movement towards microcolony interiors, then phage adsorption of these bacteria would be expected to be encouraged by microcolony bacteria rather than intentionally bypassed. (5) With reduced metabolic activity, then reduced infection vigor may be manifest via less specific or otherwise not genetically encoded means, resulting in some cases in phage burst sizes reduced to zero (effectively abortive infections) or phage infection progression which is reduced to the point that bacteria survive (effectively representing restrictive infections). In addition, it is conceivable that some genetically encoded resistance mechanisms may be enhanced in less metabolically active bacteria, e.g., with burst size reductions being more pronounced. (6) Phage adsorption to bacteria that are already infected with the same phage type will result, effectively, in the loss of one of the two (or more) phages, that is, since bacteria generally at best will support only a single phage burst. (7) Biofilm matrix material to a degree may interfere with virion diffusion, and it is conceivable that this interference is enhanced in the course of EPS maturation, i.e., as associated with *less* recently replicated bacterial cells.

Thus, we can imagine that microcolony walls must start out sufficiently thick within sufficiently large microcolonies to allow sufficient time for development of reasonably large numbers of dispersing cells. Initially too-thin walls, by contrast, could be prone to premature breaching. As considered subsequently, thick and otherwise structurally intact microcolony walls could serve not just as shear-resistant containers of developing dispersal cells, but also could effect delays in phage access to the cells found within these “containers”. The various potential mechanisms of resistance to phage penetration to the interior of microcolonies, and thus resistance to phage penetration to dispersing bacteria forming there, are summarized in Figure 6 and discussed in the following two subsections (6.2.2. and 6.2.3.).

#### 6.2.2. Sorptive scavenging

Protection of centrally located, developing seeding-dispersal cells could be afforded by a number of mechanisms. These include more mature biofilm matrix in association with older wall-layer cells, and/or less physiological active cells also in this wall layer [22]. To be most effective as barriers to phage propagation, these wall-layer cells would need to be both relatively densely packed together and reasonably receptive to phage adsorption (Figure 4). In one estimate of how tightly packed biofilm cells can be, Hamilton [133] describes a biofilm found in an oil-field pipeline that is up to 150 µm thick and containing 5 × 10^7^ cells per square centimeter, which corresponds to a bacterial density within the biofilm itself of at least 3.3 × 10^9^ cells/ml [= (5 × 10^7^ cells/10^8^ µm^2^) × (10^12^ µm^3^/ml) × (1/150 µm)].

What is known as the mean free time for a phage virion, that is, average time until adsorption for a population of phages, can be estimated as 1/*kN*, where *k* is the phage adsorption rate constant and *N* bacterial density [134]. This mean free time may be extended given slowing of phage diffusion rates such as due to the presence of EPS. It should be noted, however, that presumably EPS would slow all virion diffusion, not just *to* bacteria but also *past* bacteria. That is, phage diffusion-inhibiting EPS should slow virion diffusion not just within microcolonies but *through* microcolonies as well. Reductions in *k* due to reduced rates of virion diffusion thus should not translate into greater phage abilities to diffuse past bacteria but instead simply into lower rates of virion movement overall.

If we were to assume even a very modest adsorption rate constant, e.g., of 3 × 10^−10^ ml/min [135],[136], which is about ten-fold slower than that estimated for phage T4 to *Escherichia coli* in broth culture [137], then that would still imply a mean free time of only 1 min given 3.3 × 10^9^ cells/ml. Given that the greater the concentrations of bacteria, the faster phages are expected to adsorb (Section 3.6.), then biofilms should have at least *sufficient* bacterial densities to result in the adsorption of associated phage virions with high likelihood.

Just as outer-layer biofilm bacteria can serve as scavengers of molecular oxygen, thereby depriving inner cells of oxygen [129], so too should more external biofilm-bacteria potentially serve as draws to phage adsorption [9]. The consequence would be to slow rates of phage movement [138], such as movement towards the centers of microcolonies. Sufficiently high phage adsorption rates in particular should make rates of phage penetration into microcolonies particularly dependent on the duration of resulting phage infections [139],[140]. That in turn would serve to place a premium on infection vigor in determining these rates of penetration, versus dominance instead by virion diffusion rates. Wall cells thus could act as targets for virion “sorptive scavenging” [141] or—from a slightly different perspective—serve as virion adsorptive decoys, that is resulting in phage adsorption of somewhat dispensable, protective wall cells rather than more reproductively able, developing dispersal cells (i.e., as analogous to as discussed in Section 2.2.). The result, given sufficiently reduced wall-bacteria metabolic rates, could be a substantial slowing of phage propagation towards microcolony interiors relative to rates that might be achieved via virion diffusion alone. This slowing, that is, would be in comparison to rates of virion movement through microcolonies as could occur given, instead, an inability of virions to adsorb biofilm bacteria [9],[94].

This mechanism of sorptive scavenging explicitly would be rather than wall cells *avoiding* phage adsorption, and thereby avoiding self-sacrifice (Section 2.2.) as resulting from phage infection. As certain phage receptors may be down-regulated under more crowded conditions [142], or bacteria target sizes may be reduced given lower metabolic activity [66], we nevertheless can anticipate that microcolony bacteria will not, under all circumstances, serve as maximally effective adsorptive decoys. As noted near the top of this section, however, even with relatively low phage affinities for bacteria, as indicated by the low adsorption rate constant used in the calculation, phage mean free times may still be quite short in the biofilm environment. At a point where adsorption rate constants (*k*) are reduced so far that virion mean free times are not reasonably short within microcolonies, then presumably microcolony cells, including dispersal cells formed in the course of central hollowing, would serve more generally as less adsorbable targets.

#### 6.2.3. Secondary adsorption

In a form of “self-shading”, parasites encounter already parasite-infected hosts [143], with “self” in this case referring to an equivalent parasite. With phages, as parasites of bacteria, encountering another phage can result in virion inactivation due to phage adsorption of already phage-infected bacteria (secondary adsorption). Because bacteria generally can support only a single phage burst, these “secondary” virions will in effect tend to be “ecologically” inactivated (one infection producing, that is, at best one burst), and this “ecological” inactivation will occur whether or not primary phages display specific anti-superinfection mechanisms, such as superinfection exclusion or superinfection immunity [71]. The result would be a reduction in the effective burst sizes of phage infections [25],[26], where “effective burst size” is the number of phage progeny which go on to successfully infect new bacteria, a.k.a., the phage reproductive number [27],[28]. Reductions in effective burst sizes, that is, are a result of inactivation of infection-released virions. This inactivation in particular should continue for as long as phage-infected bacteria remain virion adsorbable, thus extending the duration, potentially to the entire pre-lysis length of phage infections, that individual bacteria can contribute to sorptive scavenging.

#### 6.2.4. Phage motivation of bacterial escape

A further consideration is whether phages, given an ability to lyse bacteria and/or to depolymerize EPS, could weaken walls containing seeding dispersal cells, that is, as these cells develop in the course of central hollowing. The idea here is that bacterial escape in this case occurs through mechanical failures (breaches) in these walls (Section 6.2.1.). If phages can accelerate wall weakening, such as by lysing component bacteria, then they may also accelerate wall failure. Wall failure should allow motile bacteria to escape from these central regions of microcolonies, and potentially do so prior to phages reaching and then propagating through what otherwise would be a metabolically active but physically contained bacterial populations. In other words, the same mechanisms which could serve to allow phages to gain access to the centers of hollowing microcolonies might also foster sooner bacterial escape from those centers.

This assertion comes with the caveat that escape presumably would need to occur through the same wall breaches through which phages can gain access. Smaller phage burst sizes, i.e., as a consequence of reduced infection vigor, or larger diameters of wall breaches, may however reduce the phage potential to acquire bacteria prior to or during this escape. That is, the phage capacity to instantaneously acquire an entire population of susceptible bacteria, even if those cells are both highly adsorbable and closely associated, will be limited by local phage densities (Section 3.6.) and thereby by effective burst sizes. Thus, very large bust sizes, if they can give rise to high local phage numbers or densities, should result in higher likelihoods of rapid phage acquisition of large numbers of local bacteria. This potential will decline, however, to the extent that neither local phage numbers nor local phage densities can reach high levels at the same moment as the breach occurs (with wall breaks potentially occurring in this scenario as a combination of prior phage-induced wall damage and damage-potentiating shear forces imposed on microcolonies by overlying fluid flow). Again (Sections 1. and 4.4.), one can envisage a race between localized phage acquisition of bacteria and bacterial escape from the vicinity of phages, with more rapid bacterial escape or lower phage numbers or densities favoring bacterial escape while the converse should favor instead bacterial acquisition by phages.

### 6.3. Protection of passive dispersal

Rates of release of bacteria, even from young biofilms, can be quite high [57]. Thus, an alternative possibility to protection of inner “nurseries” of actively forming dispersal cells, as in the course of microcolony central hollowing, instead is protection of to-be-passively released cells found especially on the surface of microcolonies (Section 3.3.). Locations along the surface of a microcolony which have not yet become phage infected thus in principle could at some point detach as single or instead as multiple cells, resulting in passive dispersal (Section 2.4.1.). In this case, mechanisms which only slow either virion movement or phage propagation may help to at least temporarily contain phages in one portion of a microcolony versus another. The result could be delay in phage translocation, translocation that is from the original point of phage encounter with a microcolony to other locations from which cell dispersal may passively occur. In general terms, at least four mechanisms could serve to impose or extend such delays. A scenario involving passive release due to abrasion is presented as well.

#### 6.3.1. Limitations to microcolony “burrowing”

The first such mechanism is as considered in the previous section (6.2.), and that is that phage propagation through microcolonies may be inefficient, especially through older, larger, or more physiologically heterogeneous microcolonies. This would suggest that phage movement from one location on the surface of a microcolony to another location on the surface of the same microcolony may occur either predominantly or more rapidly along the surface of the microcolony rather than following phage “burrowing” more deeply into the microcolony (Figure 1).

#### 6.3.2. Limitations to diffusion over the surface of microcolonies

This second mechanism derives from the potential for surface-released virions to diffuse large distances, i.e., multiple cell lengths along the surface of a microcolony, with this potential not necessarily substantial. Instead, microcolony bacteria which are immediately adjacent to released virions will likely serve as virion sorptive scavengers (Section 6.2.2.), that is, should those virions initially diffuse close to the surfaces of parental microcolonies. Such localized adsorption would be versus forming less closely associated but nevertheless microcolony-enveloping plumes. Indeed, multiple limitations likely exist on the formation of such plumes: (i) sorptive scavenging by adjacent bacteria (which would limit the number of virions available to form plumes); (ii) tendencies for virions to diffuse into the larger environment rather than back towards the parental microcolony after traveling substantial distances (again, multiple cell lengths), as indicated approximately as point (B) (1) in Figure 1; and (iii) fluid flow away from the microcolony, i.e., illustrated as point (B) (2) in Figure 1, which would have the effect of moving virions away from microcolonies faster than they would move away via diffusion alone.

The concept of “plumes” is also used by Doolittle et al. [62], probably with roughly the same connotation of “plume” as used here, with its possibility apparently similarly rejected. Namely, Doolittle et al. appear to be pessimistic about the potential for phage movement, as driven primarily by virion diffusion over longer distances, to result in phage adsorption of biofilm bacteria. Such plume formation is, also according to Doolittle et al., versus more “plaque”-like phage population growth. The latter presumably involves diffusion that tends to be more limited, that is, to towards what, following bacterial lysis, were more close-by bacteria, which Doolittle et al. suggest phage spread within biofilms “more closely resembled” (p. 337). Indeed, Doolittle et al. [62] in particular suggest that (p. 338, emphasis added), “Upon lysis of infected cells at the surface of the biofilm, the fluid boundary layer above the biofilm would tend to retain progeny phage. These progeny phage would in turn infect other biofilm cells. Hence, *cells deeper in the biofilm would most likely become infected with greater frequency upon release of progeny phage* from the initially infected cells.” Overall, I interpret this and other statements by Doolittle et al. as being *contrary* to phage virions diffusing away from biofilm bacteria to generate biofilm-enveloping plumes, plumes that then can result in virions diffusing, with high likelihood, back towards a biofilm to effect subsequent biofilm adsorption, but adsorption in a somewhat different location.

Virions released by phage-infected microcolony-surface bacteria thus should readily infect adjacent microcolony-surface bacteria (see Section 3.3. for discussion of the conception of biofilm “surface”). The likelihood of virion adsorption to the same microcolony, however, should decline the further virions are able to diffuse, as diffusion near to the microcolony surface should mostly result in close by sorptive scavenging while diffusion away from the microcolony surface should not, with high likelihood, result in virion return to the microcolony surface. The potential for relatively unimpeded virion diffusion over multiple cell lengths along the same microcolony's surface, which is then followed by infection of relatively distant microcolony surface bacteria, consequently may not be high. Due to higher metabolic rates displayed by biofilm surface bacteria versus “cells deeper in the biofilm” (Section 6.1.), there nonetheless should, despite as may be interpreted from the statement of Doolittle et al. (previous paragraph), be more rapid phage *propagation* across the surface of microcolonies (or biofilms) versus in the course of microcolony “burrowing” (Section 6.3.1.). This surface propagation thus should tend to progress with preference to infection of bacteria which are more adjacent to lysing bacteria rather than infection of more distant bacteria, though this effect may be reduced if the total volumes surrounding microcolonies are sufficiently constrained that enveloping virion plumes in fact can form.

#### 6.3.3. Longer distances as impediments to relative phage movement

The third mechanism is simply microcolony size. The greater the distances that must be traversed to reach all bacteria found on the surface of microcolonies, then the longer it should take phages to reach all of those bacteria. This should especially be the case to the extent that larger microcolony size further interferes with the ability of released phages to build up concentrated plumes of virions that are able to envelope entire microcolonies. Thus, greater microcolony size alone could contribute to delays in phage movement to other locations found on the surface of the same microcolony, as indeed greater microcolony thickness should amplify delays in phage propagation across the full width, *into* microcolonies.

#### 6.3.4. Reduced infection vigor

Given sufficient distances for phages to travel along microcolony surfaces to would-be escaping bacteria, along with both limitations on phage ability to traverse these distances via diffusion alone (Sections 6.3.2. and 6.3.3.) and inherent difficulties for phages to propagate through the interiors of microcolonies (Section 6.3.1.), then phage movement may be further delayed given reduced infection vigor. This would be as imposed via less-effective expression of bacterial resistance mechanisms such as probabilistically acting abortive infection systems since surface bacteria otherwise would be expected to be relatively metabolically active (Section 6.1.). Even absent such dedicated phage-resistance mechanisms, however, the phage ability to rapidly infect all of a microcolony surface may, as noted, be limited by other, less dedicated mechanisms, including the relative slowness—in comparison with diffusion processes—of even relatively rapid lytic cycles (Section 6.1.).

#### 6.3.5. Abrasion of released bacterial clumps

Phage-associated mechanical weakening of microcolonies at their surfaces might result in accelerated passive erosion of bacteria, perhaps as equivalent to wall weakening as considered in Section 6.2.4. Such phage-induced weakening of mechanical connections between microcolony bacteria likely would be greatest close to points of *resulting* bacterial escape, thereby necessitating that bacterial erosion occur prior to associated phage adsorption in order for such otherwise passive escape of bacteria from phages to be successful. This balance might be difficult to achieve, however, unless phage-induced mechanical weakening propagates across microcolonies faster than phage virions themselves. Alternatively, with or without phage input, clumps of bacteria may be released (Figure 2; Section 2.4.). To the extent that these clumps subsequently release cells, such as due to abrasion in the course of encounter with surfaces, then bacteria, given delay in phage propagation through these clumps, may be less likely to be phage infected at the point of their release. This could allow successful passive bacterial escape even if clumps as a whole are not altogether free of phages.

## 7. Ecological Rather than Necessarily Evolutionary Relevance of Delay Mechanisms

The various mechanisms considered in sections 6.2. and 6.3. (Figures 1 and 6) may be useful only to the extent that phage numbers within environments are sufficiently low that phage encounters with microcolonies are relatively rare events (Section 5.3.). For example, microcolony encounter, over the span of its existence, with only on average roughly a single phage to which it is susceptible may be enough to select for mechanisms contributing to phage delay, and particularly so long as such delay results in meaningful increases in the number of bacteria which succeed in escaping. Alternatively, microcolony encounter frequencies approaching one phage per *cell* should substantially reduce advantages associated with delay alone. The tipping point between circumstances which may or may not advantage bacteria in terms of delaying phage propagation through microcolonies, however, presumably depends on the specifics of different systems.

Delay mechanisms, though, may be present for purposes or as phenomena that exist for reasons other than countering attacking phages (Section 3.1.). These include formation of nutrient gradients (Section 6.1.), microcolony structural support (Section 6.2.1.), fluid dynamics surrounding microcolonies (Section 6.3.2.), microcolony size (Section 6.3.3.), and perhaps even selection for more complete phage protection but against different phages (Sections 4.2. and 4.3.). Mechanisms of delay thereby, at least in part, could serve as extensions of these existing utilities. Mechanisms of phage delay as a consequence may not necessarily be maintained by biofilm bacteria specifically as defenses against a given type of attacking phage but nevertheless could serve as means by which biofilm bacteria can increase the breadth of phages to which they can display at least some resistance (Section 4.3.). Delay mechanisms may be better viewed, therefore, as contributing to phage-biofilm *ecological* dynamics rather than as mechanisms which would exist only given ongoing selection for imperfect resistance to specific phage types. In other words, appreciating mechanisms of phage delay may be more important towards understanding the ecology of phage-biofilm interactions rather than serving as phenomena of substantial evolutionary interest. Included among this ecology of phage-biofilm interactions may be the applied ecology of phage therapy.

## 8. Phage Therapy of Chronic Bacterial Infections

Chronic bacterial infections are typically associated with biofilms [45],[123],[124],[125]. Phages, in the guise of phage therapy [128], have been proposed as possible alternatives to conventional antibiotics to treat especially antibiotic-resistant bacterial infections. Antibiotics, however, remain as first-line defenses against these bacteria. Consequently, by the time alternative approaches such as phage therapy are invoked, these infections often have become chronic, sometimes for years, resulting in treatments involving phage application towards clearance of what presumably are mature bacterial biofilms. Though the bacteria making up these biofilms may not be phage resistant, in a conventional sense of outright blocking phage propagation as may be measured *ex situ*, they nevertheless presumably can display less robust resistance mechanisms. That is, they may still effect delays on phage propagation which thereby serve as at least partial impediments to the removal of these biofilms. Furthermore, animal models of presumptive chronic bacterial infections as yet do not necessarily effectively replicate these properties [144]. In what way may simply a better appreciation of delay mechanisms therefore help to inform treatment approaches?

In many ways the answers to that question may already have been addressed as a result of trial and error in the clinic [121],[126],[128],[145]. Most notable is removal of biofilm via infection debridement prior to phage application, which presumably increases the phage potential to reach targeted bacteria. In addition is the application of phages in multiple doses. Multiple dosing presumably makes up for less-than-robust phage abilities to boost their own numbers *in situ*, i.e., as due to reduced vigor of phage infections taking place within more mature biofilms—biofilms, that is, which possess bacteria with stationary phase-like physiologies. In short, to the extent that phages propagate through mature biofilms on their own too slowly or too inefficiently, then first reducing bacterial numbers by other means (debridement) and otherwise taking advantage of the phage potential to at least reach and eliminate biofilm-surface bacteria (Section 3.3.) may serve to expose more inner biofilm layers of bacteria to phages. If phage infections lack necessary vigor to produce sufficient phage offspring to effectively target all newly exposed and/or metabolically revived bacteria, then dosing with additional phages presumably can make up for that deficiency.

This issue of capacity to infect with sufficient vigor may also be addressed prior to the commencement of phage therapy by determining the potential for a phage to produce plaques of reasonably large size and with reasonable efficiency of plating. Alternatively, and potentially providing improved characterization, is demonstration of phage ability to display reasonably large burst sizes, reasonably short latent periods, and reasonably high rates of adsorption [146], perhaps including with late-log phase bacteria serving as hosts. That is, to employ approaches to phage characterization other than solely demonstrating a potential to form “spots” (zones of inhibition) on bacterial lawns, as spots typically supply minimal information regarding phage ability to robustly infect a given bacterium [147]. It is conceivable also that bacterial lysis could induce or accelerate bacterial escape from biofilms (Section 6.2.4.), while debridement could otherwise stimulate new biofilm growth. By sustaining consistently high phage numbers *in situ* through multiple or continuous dosing [148], then newly released planktonic cells, metabolically revived biofilm bacteria, or new biofilm growth—as may indeed be more susceptible to phage attack than more mature biofilm material [22]—may be targeted and eliminated.

There thus likely exist multiple mechanisms of biofilm resistance to phage infection, many of which are not necessarily able to outright block phage propagation. By being conscious of the potential of biofilms to interfere with the therapeutic performance of phages, however, we can at least make informed decisions as to how treatment protocols may be improved. Especially, we may consider (i) infection preparation towards increasing the availability of bacteria to phages, (ii) increasing the frequency of phage application, and indeed (iii) simply being aware that treatments overall may take relatively long periods of times to be effective. For example, weeks to overcome inherent delays imposed by biofilm bacteria on phage antibacterial impact. Improved animal models of chronic bacterial infections towards explicitly studying the utility of such practices likely would be useful as well [144].

## 9. Conclusion

Elsewhere I have explored phage-biofilm interactions from the perspective of phage potential to survive and prosper ecologically [8],[21]. Considered as well is the phage potential to fully exploit bacterial microcolonies particularly during phage plaque formation [20],[21],[23]. Here I have taken a more detailed look at this latter potential, particularly from the perspective of phage-attacked bacteria rather than from that of attacking phages [22]. I suggest in particular that bacteria likely possess more mechanisms of phage resistance within biofilms than we may normally consider in terms of more traditional phage-resistance mechanisms and that these mechanisms may vary in their effectiveness as well as utility both temporally and spatially within biofilms. In addition, ultimately what biofilm bacteria are protecting with these mechanisms is the potential for phage-uninfected bacteria to escape to new and, ideally for escaping bacteria, relatively phage-free locations. The presented scenarios by necessity are speculative but nevertheless draw on current understanding of biofilm, bacteria, and phage biology. Ultimately, it likely is important to consider phage-biofilm interactions as a diversity of scenarios even when limiting considerations to specific phage, bacteria, biofilm types, and conditions. One area in which such considerations can have applied relevance is in the course of phage-mediated biocontrol of bacterial biofilms such as during the phage therapy of chronic bacterial infections.
